# Cognitive remediation in schizophrenia: the earlier the better?

**DOI:** 10.1017/S2045796019000532

**Published:** 2019-09-26

**Authors:** Marcella Bellani, Chiara Ricciardi, Maria Gloria Rossetti, Niccolò Zovetti, Cinzia Perlini, Paolo Brambilla

**Affiliations:** 1 Department of Neurosciences, Section of Psychiatry, Biomedicine and Movement Sciences, University of Verona, Verona, Italy; 2 Department of Neurosciences and Mental Health, IRCCS Ca'Granda Ospedale Maggiore Policlinico, Milan, Italy; 3 Department of Neurosciences, Section of Clinical Psychology, Biomedicine and Movement Sciences, University of Verona, Verona, Italy; 4 Department of Pathophysiology and Transplantation, University of Milan, Milan, Italy

**Keywords:** Cognition, cognitive remediation, early schizophrenia, neuroimaging, review

## Abstract

Impairments in neuro and social cognition are considered core features of schizophrenia (SCZ) since they affect patients' functioning and contribute to poor socio-occupational outcomes. Therefore, the improvement of cognitive performances has become a primary goal in the care of patients with SCZ, especially in the first phases of the disease, as early interventions may favour better long-term outcomes. Cognitive remediation (CR) is a behavioural training aimed at improving cognitive functions with the goal of durability and generalisation in everyday life. Neuroimaging studies suggest that CR leads to neuroplasticity in chronic SCZ, whereas only a few studies tested the neural effects of CR in the early phase of the disease. Thus, in this review, we aimed at summarising CR-induced structural and functional brain changes in early SCZ. Existing evidence showed a protective effect of CR on grey matter volume in selected medial-temporal (i.e. hippocampus, parahippocampus and amygdala) and thalamic regions whereas functional changes affected mostly dorsolateral prefrontal and insular cortices both associated with improvements in cognitive performance and emotion regulation. Overall, CR in early SCZ appears to be associated with neural adaptations mostly allocated in prefrontal and limbic regions, however future longitudinal studies are needed to clarify whether the positive effects of cognitive training persist over time. It may also be interesting to investigate whether the application of CR in the early *v*. the late stage of the disease may lead to incremental benefits.

Schizophrenia (SCZ) is a chronic psychiatric disorder characterised by marked cognitive impairments that affect functional ability, predict poor interpersonal and socio-occupational outcomes and appears associated with structural and functional brain changes in this population (Green *et al*., 2004; Barch and Ceaser, 2012). Since cognitive deficits of patients with SCZ respond only moderately to pharmacotherapy, in the last 15 years, researchers have focused on behavioural interventions aimed at improving the patient's functioning in everyday life such as cognitive remediation (CR) (Wykes *et al*., 2011). A growing number of studies showed durable effects of CR on cognition and global functioning as well as a positive effect on neural activity of patients with chronic SCZ (Wykes *et al*., 2011; Penadés *et al*., 2017). Conversely, only a paucity of neuroimaging studies investigated the effect of CR in early SCZ and results are still sparse (Eack *et al*., 2010, 2016; Keshavan *et al*., 2017; Ramsay *et al*., 2017). Given that brain changes occurring in SCZ appear to worsen over time (Torres *et al*., 2016), the application of CR in the early phase of the disease may play a crucial role in protecting against future neural alteration and its subsequent impact on cognitive abilities and functioning.

In this review, we aimed to describe neuroimaging studies that investigated the effect of CR on brain structures and activity in patients with SCZ in the early phase of the disease.

The bibliographic search was performed using PUBMED and Web of Science databases. The following keywords were used for the search: (cognitive) AND (remediation OR training OR rehabilitation) AND (early OR first episode) AND (psychosis OR schizophrenia) AND (magnetic resonance imaging). The inclusion criteria are: (i) original publication published in a peer-reviewed journal, (ii) English language, (iii) the use of a structured protocol for CR training, (iv) the inclusion of a comparison group undergoing a control therapy and (v) application of structural or functional neuroimaging techniques. After title and abstract screening, four studies were identified and included in the review, three of which used the same cohort of patients (Eack *et al*., 2010, 2016; Keshavan *et al*., 2017). Methodological characteristics and main findings from each study are shown in Table 1.

**Table 1.** Neuroimaging studies of CR in early SCZ

Reference	Number of participants (M/F)	Age (mean ± s.d.)	Years of illness (mean ± s.d.)	Diagnosis (*n*)	CR intervention	Intervention length	Imaging method	Main results
Ramsay *et al*. (2017)	CR: 43 (31/12) CT: 43 (33/10)	21.70 ± 3.26 20.74 ± 3.37	1.57 ± 1.30 1.68 ± 1.45	SCZ (ns) SAD (ns)	AT	40 h/8–10 weeks	sMRI (3T)	CR *v*. CT showed larger GM thalamic volumes after the training. L thalamus GM volumes correlated positively with cognitive performance. Findings suggest that the cognitive gains induced by CR are associated with structural neuroplasticity in the thalamus.
Keshavan *et al*. (2017)	CR: 25 (14/11) CT: 16 (12/4)	24.95 ± 5.16 26.13 ± 5.29	3.36 ± 2.45 2.97 ± 1.84	SCZ (26) SAD (15)	CET	60 h/3 months ( + 45 weekly sessions of social-cognitive group)	fMRI (3T)	CR *v*. CT showed increased activity in the R dlPFC activation and reduced connectivity between dlPFC and ACC. Increased R dlPFC activity correlated positively with neurocognitive improvements.
Eack *et al*. (2016)	CR: 25 (14/11) CT: 16 (12/4)	29.95 ± 5.16 26.13 ± 5.29	3.36 ± 2.45 2.97 ± 1.84	SCZ (25) SAD (16)	CET	60 h/3 months ( + 45 1.5 h weekly sessions of social-cognitive group)	rsfMRI (3T)	CR *v*. CT showed greater functional connectivity preservation between the resting-state networks involved in emotion processing and problem solving (i.e. fronto-temporal network) and the L dlPFC and increased connectivity between the fronto-temporal network and the R insula. In patients treated with CR increased insular and L dlPFC connectivity correlated with improved emotion perception and regulation, respectively.
Eack *et al*. (2010)	CR: 31 (ns) CT: 27 (ns)	26.17 **±** 6.51	3.22 **±** 2.2	SCZ (53) SAD (18)	CET	60 h/3 months ( + 45 1.5 h weekly sessions of social-cognitive group)	sMRI (3T)	CR *v*. CT showed greater preservation of GM volume in the L hippocampus, parahippocampal, fusiform gyrus and L amygdala GM volume increases. Volumetric changes were associated with improved cognition.

ACC, anterior cingulate cortex; AT, auditory training cognitive remediation; BOLD, blood-oxygenation level dependent; CET, cognitive enhancement therapy; CR, cognitive remediation; CT, control therapy; dlPFC, dorsolateral prefrontal cortex; F, females; fMRI, functional magnetic resonance imaging; GM, grey matter; L, left; M, males; ns, not specified; R, right; SCZ, schizophrenia; SAD, schizoaffective disorder; rsfMRI, resting state functional magnetic resonance imaging; sMRI, structural magnetic resonance imaging; T, tesla.

Ramsay *et al*. (2017) conducted a structural magnetic resonance imaging (MRI) study to test the effect of a CR training *v*. a control therapy, on grey matter volumes of patients with early SCZ (Ramsay *et al*., 2017). After 40 h of training, the CR group showed larger thalamus than the control group. Moreover, within the CR group, left thalamus correlated positively with changes in cognitive performance (Ramsay *et al*., 2017). Additional three neuroimaging studies were conducted in early SCZ, by using the same sample of patients randomly assigned to a CR training (i.e. cognitive enhancement therapy –CET), or enriched supportive therapy (EST) and treated for two years (Eack *et al*., 2010, 2016; Keshavan *et al*., 2017). In the first study by Eack *et al*. (2010) patients undergoing CET showed greater preservation of grey matter volume over the course of 2 years in the left hippocampus, parahippocampal gyrus and fusiform gyrus, and greater grey matter increases in the left amygdala than those receiving EST. Lower grey matter loss in the left parahippocampus and fusiform gyrus was significantly associated with improved neuro and social cognition, while greater grey matter increases in the left amygdala was associated with improved social cognition suggesting that amygdala may play a key role in social-cognitive processes (e.g. perspective-taking) (Lamm *et al*., 2007).

In a second study, Eack *et al*. (2016) examined the effects of CET on frontotemporal brain connectivity, using resting-state functional MRI (Eack *et al*., 2016). Compared to the control group, patients treated with CET showed lower reduction of the left dorsolateral prefrontal cortex (dlPFC) connectivity and increased right insula connectivity with the resting state network. Moreover, within the CET group, increases in the connectivity between the right insula and the left dlPFC were associated with improvements in emotion perception and regulation, respectively (Eack *et al*., 2016).

In the third study, the authors aimed at exploring longitudinal changes in brain activations and connectivity in the CET *v.* EST group, and the association with performances at cognitive tasks (Keshavan *et al*., 2017).

Patients treated with CET exhibited a continuous increase of neural activity in the right dlPFC, that was associated with moderate improvements in neurocognition, suggesting a potential neuroprotective effect of CET. By contrast, patients treated with EST revealed a progressive reduction of task-related neural activation. Moreover, functional connectivity analysis showed decreases in connectivity between the dlPFC and the anterior cingulate cortex (ACC) in CET compared to EST over the two years of treatment, which was associated with neurocognitive improvement. Conversely, patients treated with EST showed no changes in functional connectivity over time (Keshavan *et al*., 2017).

Overall, available MRI studies in patients with early SCZ suggest that CR interventions are associated with structural and functional brain changes mostly allocated in frontal and limbic regions (i.e. hippocampus, amygdala, dlPFC and ACC) (Eack *et al*., 2010, 2016; Keshavan *et al*., 2017; Ramsay *et al*., 2017). In particular, CR appears to decelerate or partially reverse progressive brain volume deterioration occurring in the early phases of illness in areas known to be crucial for higher-order cognitive processes, including the frontal cortex, thalamus, hippocampus and the amygdala (Eack *et al*., 2010; Ramsay *et al*., 2017). Consistently, CR seems to have a ‘normalising' effect on resting-state networks and functional activity in the frontotemporal network, amygdala and ACC of patients with early SCZ (Eack *et al*., 2016; Keshavan *et al*., 2017). Moreover, neuroplastic changes associated with CR in early SCZ correlated with improvements in neuro and social cognition in all the presented studies (Eack *et al*., 2010, 2016; Keshavan *et al*., 2017; Ramsay *et al*., 2017). It is plausible that these ameliorations can be obtained by increasing or decreasing signalling in neural circuits (Penadés *et al*., 2017).

Even though CR effects in early SCZ resemble those observed in chronic patients, we can speculate that the neuroprotective effect of CR could be the highest the earliest the intervention is offered, as in the early phase of illness neurodevelopmental processes are still taking place (Corbera *et al*., 2017). Moreover, early interventions proved to be effective in reducing negative symptoms and increasing socio-occupational functioning in SCZ (Bond *et al*., 2015; Murru and Carpiniello, 2018). Thus, given the efficacy of early interventions in other domains of SCZ, it is also plausible that intervening on cognitive impairments in the early stages of SCZ may lead to a better long-term functional outcome and possibly have a protective effect against neural alterations associated with the pathology.

To conclude, CR appears to be a promising approach in the treatment of cognitive deficits and neural alterations associated with the early phase of SCZ. However, evidence is still sparse, as only a paucity of studies has been conducted with limited control for confounding factors (e.g. heterogeneous patients' populations and methodology). Particularly, the long-term effect of CR interventions on brain plasticity has not been investigated yet. As such, future studies (using multimodal techniques) are needed to assess whether CR may be considered a sustainable approach with long-lasting positive effects on cognitive and neural alterations of patients with SCZ. Particularly, we warrant the conduct of longitudinal studies starting from the very early stage of the illness to clarify whether the application of CR in the early *v*. chronic phase of the disease may have incremental benefits.
